# Fabrication of a Low Density Carbon Fiber Foam and Its Characterization as a Strain Gauge

**DOI:** 10.3390/ma7053699

**Published:** 2014-05-08

**Authors:** Claudia C. Luhrs, Chris D. Daskam, Edwin Gonzalez, Jonathan Phillips

**Affiliations:** 1 Mechanical and Aerospace Engineering Department, Naval Postgraduate School, 700 Dyer Rd., Monterey 93943, CA, USA; E-Mail: dcdaskam@nps.edu; 2 Hartnell College, Salinas, CA—Naval Postgraduate School, Monterey 93943, CA, USA; E-Mail: eigonzal@nps.edu; 3 Physics Department, Naval Postgraduate School, 833 Dyer Rd., Monterey 93943, CA, USA; E-Mail: jphillip@nps.edu

**Keywords:** carbon nanofiber, viscoelastic, strain gauge, low weight, porous, electrically conductive, hydrophobic

## Abstract

Samples of carbon nano-fiber foam (CFF), essentially a 3D solid mat of intertwined nanofibers of pure carbon, were grown using the Constrained Formation of Fibrous Nanostructures (CoFFiN) process in a steel mold at 550 °C from a palladium particle catalysts exposed to fuel rich mixtures of ethylene and oxygen. The resulting material was studied using Scanning Electron Microscopy (SEM), Energy Dispersive Spectroscopy (EDX), Surface area analysis (BET), and Thermogravimetric Analysis (TGA). Transient and dynamic mechanical tests clearly demonstrated that the material is viscoelastic. Concomitant mechanical and electrical testing of samples revealed the material to have electrical properties appropriate for application as the sensing element of a strain gauge. The sample resistance *versus* strain values stabilize after a few compression cycles to show a perfectly linear relationship. Study of microstructure, mechanical and electrical properties of the low density samples confirm the uniqueness of the material: It is formed entirely of independent fibers of diverse diameters that interlock forming a tridimensional body that can be grown into different shapes and sizes at moderate temperatures. It regains its shape after loads are removed, is light weight, presents viscoelastic behavior, thermal stability up to 550 °C, hydrophobicity, and is electrically conductive.

## 1. Introduction

Carbon nanotubes, in particular, as single wall tubes (SWCNT), have been demonstrated to present remarkable response as piezoresistive elements [1–4]. Individual tube gauge factors, which can be described as the sensitivity of the sensor, have been reported to reach values up to 1000 [5]. However, the sensors based on CNT assemblies have much lower sensitivities than those of the individual tubes mentioned above, typical ensemble piezoresistive sensor have gauge factors up to 22 [6]. Obtaining the maximum possible gauge factor is limited by the features that dominate large assemblies of tubes: defects and difficulties controlling the orientation and position of the tubes to produce homogenous large samples. Thus far, efforts in this direction have opted for using random assemblies of individual tubes, both as single and multiwall tubes [7–10]. Thus, new materials that can attain the expected electrical and mechanical characteristics, or novel approaches to develop such materials with high yields and repeatable results, are still needed.

Other issues limiting the application of carbon structures for pressure/strain gauges are the unresolved engineering challenges. Earlier reported strategies to develop pressure/strain sensors using carbon nanotubes or carbon fibers are quite complex, difficult to reproduce and expensive. Control over the macroscopic object geometry, density, and means to create an interface with other components has turned to be a titanic effort. Some examples found in recent publications to generate three-dimensional carbon tube/fiber based architectures involve the association of carbon structures with polymeric matrices [11–13], strategies to assemble them during synthesis [14–16] and attempts to create the connections post-synthesis [17–23]. From the former category, the combination with polymers imprints undesired characteristics in the product such as reduced conductivity and low thermal stability due to the use of polymeric matrices. From the second approach: despite the products presenting the desired electrical properties, there has been no reported success in creating highly porous mechanically robust tridimensional architectures. The third approach, post-synthetic routes to generate low density structures, is dominated by techniques that use solvents to align nanotube forests, foams or cellular structures aided by the capillary forces that are present as the solvent evaporates [17,18]. Other post-synthesis efforts include low temperature soldering [19], use of monolayer and multilayered silica templates [20], high frequency pulses of electrical discharge to machine targeted shapes, and radical initiated thermal crosslinking of CNT [21,22], focused laser beam used to locally burn regions of a dense forest [23] and chemical processes to stitch CNTs together [17]. Thus, there is still a necessity for a simple, reproducible way to create mechanically robust 3D structures to be used as sensing element.

Recently it was established that macroscopic carbon foam could be fabricated in virtually any shape using the constrained formation of fibrous nanostructures process (CoFFiN) [24]. These foams consist of a solid mat of intertwined nanoscale carbon fibers, the shape of the same defined by the shape of the mold in which it is grown. The constrained formation of fibrous nanostructures process was developed as a variation on graphitic structures by design (GSD) technology [25,26]. The key to GSD, as shown in earlier work, is the catalyzed growth of solid carbon structures from radical species formed homogenously during a fuel rich combustion process. In practice, it has been clearly demonstrated that a variety of carbon structures (e.g., fibers, solid mats, graphite encapsulated catalyst, *etc.*) can be grown from combustion mixtures, and that the nature of the structure that grows is a function of catalyst, composition of the fuel rich combustion mixture, temperature, and even gas flow rate. The CoFFiN process followed to generate the carbon fiber foam studied herein is a natural extension of those previous discoveries: the catalysts, the gas mixtures, and temperatures employed, are selected to be those that lead to the most rapid growth of carbon fibers as revealed in earlier studies. Moreover; by confining growth to a mold the fibers become entangled, leading to the growth of cohesive macroscopic fiber structures that have the same shape as the mold.

In those earlier publications it was demonstrated, for CoFFiN materials, that there is a relationship between applied stress and electrical resistance, but the data was insufficient to fully characterize all features or even to clearly identify the class of material according to its mechanical behavior. The data from the present work permits the following points to be elucidated: (i) CFF on the macroscopic scale behaves precisely as a viscoelastic, and is not simply elastic; (ii) Only after repeated cyclic compression, that is “mechanical aging”, do mechanical and electrical properties stabilize; (iii) the material is not, despite earlier suggestion, particularly suited for use in a pressure gauge, instead the material is suitable for use as the sensing element in a strain gauge; and (iv) other key characteristics of the structure, such as relative density, surface area, contact angle and thermal stability are now accurately measured. Finally, it is clear that the CFF microstructure, totally composed of conductive fibers, makes it an unusual viscoelastic.

## 2. Results and Discussion

### 2.1. Results

**Figure 1.**
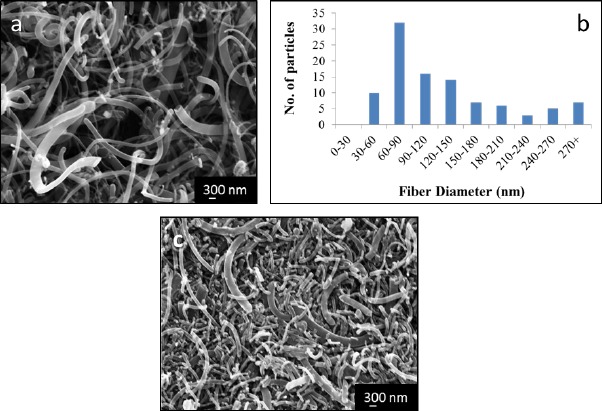
Microstructural analysis by SEM. (**a**) The sample consists of fibers which diameters vary between 30 and 400 nm and regions of empty space; (**b**) The plurality of fibers are between 60 and 90 nm in width; (**c**) During compression the empty spaces between fibers disappear with no evidence of fiber delamination or fracture.

After growth and removal from the mold, a single block of carbonaceous material was obtained. The sample roughly had the shape of the mold, although the material tended to twist modestly due to some internal stresses upon removal from the mold. Macroscopically, the product of the synthesis had the texture and consistency of foam. Examination of the sample by SEM revealed that the microstructure is dominated by intertwined fibers of diverse diameters but most of the volume is void space. Analysis of fiber diameters from electron micrographs revealed that the diameters range from approximately 30 nm to 400 nm. Within that distribution the plurality of the fibers have between 60 and 90 nm in width (Figure 1a,b). Given that the fibers grow in random directions while intertwining into each other, the length of individual fibers was not measured. Backscattered electron images of the sample showed a homogeneous distribution of the palladium particles used as catalyst. EDS analysis of the sample showed no evidence of oxygen in regions where Pd was present, indicating that despite the small amounts of oxygen used during the synthesis the palladium remains the metallic state throughout the fiber growth step.

As described in the previous section, a sample of the fiber 3D architecture was compressed inside the SEM using a SEM tester using loads up to 350 N. Micrographs taken during the experiment show that the gaps and empty spaces between the fibers decreased while under load; however, no evidence of fiber failure was detected. After the 350 N were applied the sample compressed to a point that the instrument could not place the platens any closer. Under such circumstances the sample bulged in the directions perpendicular to the applied load but regained its shape and volume after removal of the load. Indeed, even after multiple compressive cycles no breakage, delamination or cracking signatures in individual fibers were seen (Figure 1c). Study of the microstructure of the sample by SEM after mechanical cycling tests showed the same initial characteristics: intertwined fibers with gaps and void space in between them.

According to our measurements the entangled fiber structure as prepared has a relative density of 0.24, similar to cork [27] and aluminum foams [28]. Given that the density of carbon fibers is generally estimated to be the same as graphite, approximately 2.26 g/cm^3^, this indicates that approximately 90% of the volume of an uncompressed sample is void space. The BET surface area was relatively high, 118 m^2^/g. For comparison, most reported specific surface areas of graphene measured by BET range between 600 and 1000 m^2^/g [29–32], although the theoretical specific surface area has been computed to be 2630 m^2^/g [33].

**Figure 2.**
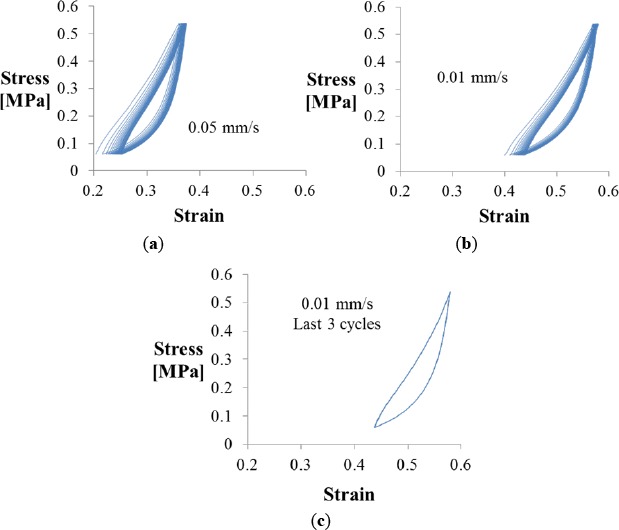
Stress *vs.* strain curves for the constrained sample. Cycles performed between 10 and 90 N loads using Plexiglas fixture to maintain constant area. The first loading cycle from 0 to 90 N has been removed. (**a**) Cycling behavior at a rate of 0.05 mm/s; (**b**) Cycling at frequencies of 0.01 mm/s; and (**c**) Values reach a reproducible and stable profile after the first conditioning 15–17 cycles.

The strain that resulted from the application of diverse loads to the fiber structure when using an Instron mechanical tester in compression mode is presented in Figure 2. Under deformation the material presents both viscous and elastic characteristics, with evidence of time-dependent values. The stress *vs.* strain curves of the constrained specimen (using the Plexiglas fixture) show the typical profile of a viscoelastic material; the first section follows a linear—elastic behavior, which is followed by an unloading cycle that does not reproduce the original stress *versus* strain path but presents instead a hysteresis loop typical of viscous materials. When cyclic loading is applied this phase lag is more evident, showing dependence with the loading or strain rate (Figure 2a,b). As other viscoelastic materials, the CFF seems stiffer when loaded fast than when subject to loading at lower rates. Each consecutive cycle performed between 10 and 90 N shows a shift in the corresponding strain to higher strain values during the first 15–17 cycles. Once conditioned the next cycles showed stable behavior, with stress strain profiles that perfectly overlap for higher number of cycles, as shown in Figure 2c. Estimation of the elastic modulus of the linear region for experiments performed at diverse strain rates demonstrate that higher frequencies produce higher values (above 4 MPa), while slower frequencies reach lower values (close to 3 MPa). The area inside the curve usually correlated to the energy absorption at a particular frequency, changes as well: the higher the cycling frequency, the lower the energy absorption capability.

**Figure 3.**
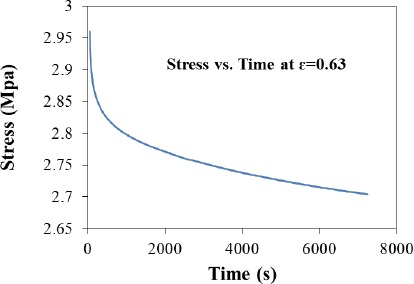
Sample relaxation. A constrained sample was maintained at a constant strain of 0.63 at room temperature. Initially 50 N were applied; the program was adjusted to maintain such strain level and stress over time recorded.

A stress relaxation test was performed by compressing the sample with an initial force of 50 N, which reduced the structure dimension by 2.5 mm, at room temperature. The sample was allowed then to relax while the position was maintained, thereby maintaining a constant strain of 0.63 on the sample. The stress on the sample was then allowed to vary and recorded. As denoted in the stress *versus* time graph upon the imposition of a constant deformation the force necessary to maintain the deformation decreases with time (Figure 3), as is typical in viscoelastic materials like biofilms and gels [34,35].

**Figure 4.**
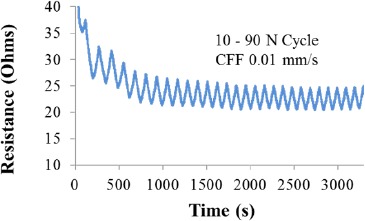
Resistance *vs.* time cyclic behavior. Cycles performed between 10 and 90 N loads using Plexiglas fixture illustrate that after some initial the conditioning cycles the Carbon Fiber Foam electrical behavior stabilizes.

The resistance of the sample can be related to the values of load and strain imposed in the 3D fiber architecture, the higher the level of load/strain the lower the sample resistance. However, the initial resistance values recorded during cyclic tests seem not to be reproducible, in the same way that mechanical tests showed with the hysteresis loop behavior, the sample requires a conditioning stage of about 15–17 cycles before the resistance values can be correlated to a particular value of strain. Figure 4 exemplifies such behavior. If resistance is plotted against load or stress, a phase lag is observed and a perfect linear correlation is never reached. However, if the correlation of resistance is made with respect to the values of strain, then a perfect line is obtained for multiple cycles. Such linear relationship between strain and resistance suggests the material could be employed as a strain gauge.

**Figure 5.**
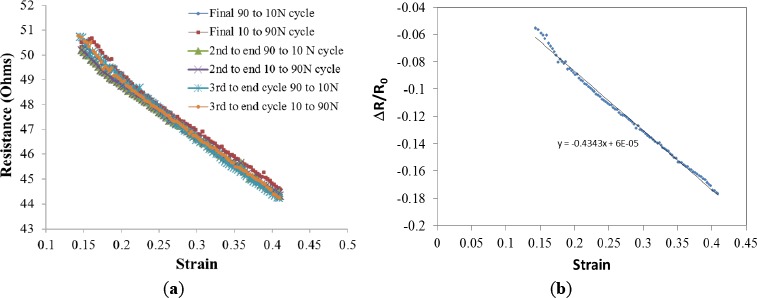
Strain Gauge. The resistance *vs.* strain values taken from the final six segments of cycling experiment show the “aged material” displays a linear relationship between resistance and strain (**a**). The slope of the (∆R − R_o_)/R_o_ *vs.* strain, taken from the 3rd to last cycle, has been used to calculate the strain gauge factor (**b**).

In order to test this proposition the resistance as a function of strain for the final six legs of the process were plotted (Figure 5a) along the ∆R/R_0_ *versus* strain (Figure 5b). The slope of the later, 0.43, is related to the sensitivity of the strain gauge or gauge factor. Despite this low value, the material does appear to have potential as sensing element of a strain gauge when high levels of strain are present.

**Figure 6.**
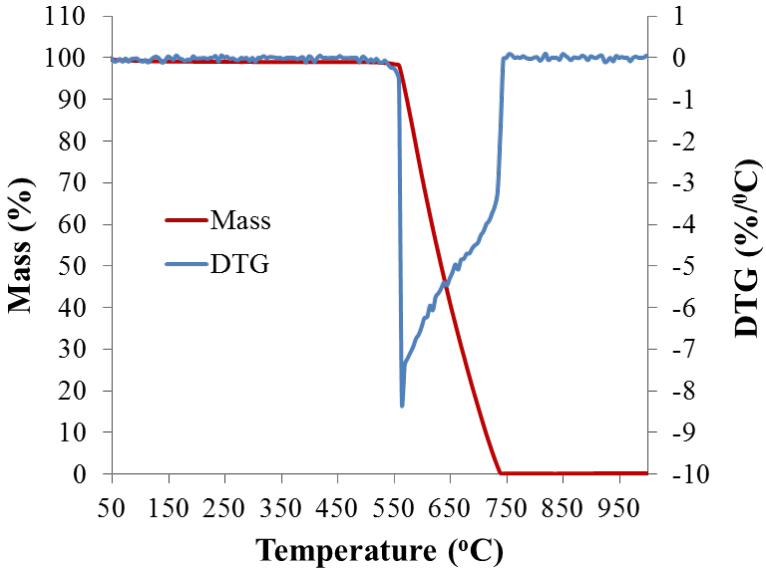
Thermal stability determined by TPO analysis. The sample maintains its weight up to at least 550 °C under an oxygen containing atmosphere, after such the carbon starts to burn off until only the weight of the original palladium catalyst particles, now oxidized, remains.

Temperature programmed oxidation analysis of the sample using 20% oxygen/80% inert gas atmospheres indicated that the sample does not suffer any weight changes until nearly 550 °C. From 550 to 750 °C the sample reacts with the oxygen to undergo a combustion reaction producing CO_2_ as volatile byproduct while losing weight until most of the sample is consumed. At 1000 °C, less than 1% of the original weight remains, associated with leftover Pd catalyst that reacted to produce PdO as single solid byproduct (Figure 6). In comparison, commercial viscoelastic polymers begin to decompose in air at above ~200 °C and are dramatically and irreversibly modified by 300 °C [36]. The CFF under study is completely stable to 550 °C during heating in air.

**Figure 7.**
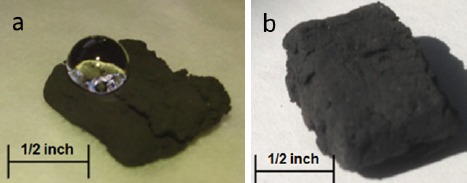
Hydrophobicity. (**a**) Drop of water suspended in the foam surface; (**b**) A drop of oil gets readily absorbed within the foam structure, showing no evidence of oil on the surface after just fractions of a second of contact.

The hydrophobicity of the samples was demonstrated by placing a water droplet on the surface of the as prepared 3D fiber structure (Figure 7a). The water droplet does not wet the foam and tends to roll off the surface at low angles of inclination. Measurement of the contact angle using the Young-Laplace method, as described in the experimental section, returned a value of 145°. In contrast, a non-polar liquid, mineral oil, did not form a drop at all, being instantly absorbed by the fiber foam (Figure 7b).

Finally, an effort was made to determine if carbon, aluminum and stainless steel could generate a similar signal than the one observed for the CFF employed. In all cases the measured resistance was in the order of 0.1 Ω. Grafoil, a form of commercial carbon created from compressed naturally occurring graphite, of approximately the same thickness as the CFF was placed in the same Plexiglas constraint system and tested using the same constant changing strain rate as that employed for the CFF, 0.01 mm/s, over the same compression limits. The general trend of resistance was the same as that observed for the CFF: resistance decreased with increased strain. However; at least for the number of cycle tests conducted herein, Grafoil resistance and strain values are extremely erratic, and do not present a linear correlation. Moreover, the material never regains its original dimension.

The primary results are briefly summarized as follows. First, mechanically stable samples of CFF clearly behave as viscoelastic material as shown by the fact that after appropriate conditioning, a repeatable hysteresis loop is observed in the stress-strain relationship. Second, there is a linear relationship between electrical resistance and strain that becomes remarkably stable after only a few cycles. The strain range over which this linear relationship is observed is very large, up to 60%. Third, relative to carbon in another form, Grafoil, CFF showed far more regularity with regard to stress/strain and resistance. Fourth, the rest of the characteristics of the sample; light weight, high surface area, hydrophobicity, conductivity, and possible energy absorption open the opportunity for using it in multiple applications.

### 2.2. Discussion

The discovery in succession over the last two decades of carbon fullerenes, carbon nanotubes [37] and the special properties of graphene [38], dramatically increased the interest in possible applications of carbon nanostructures, both on the basis of electrical and mechanical properties. For example, carbon nanotubes are under consideration for use in “molecular scale” logic circuits. That is, carbon nanotubes are seriously considered as the active elements for logic circuits, possibly replacing silicon [39–41]. There is also an enormous effort focused on using nanotubes to strengthen composite materials [42–44]. Currently there is widespread interest in employing graphene in supercapacitors due to its high surface area [32,45]. Graphene is also believed to have great potential as a component in corrosion resistant paint, light-strong plastics for cars, sports equipment, aerospace and military applications [46–48].

The present work suggests that another novel carbon material, carbon fiber foam (CFF), also has unique electrical and mechanical properties, which may lead to widespread applications. In particular, the present work provided a variety of data that help define the properties of this material, a proof of concept in evaluation of potential applications. First, application requires repeatability. We were able to duplicate the generation of the material, previously only generated in a single lab [49], and were also better define the optimal production conditions. Second, the results suggest this material can be used in place of the current generation of viscoelastic materials. Indeed, after a few cycles (*ca.* 15) the material becomes a viscoelastic with stable mechanical properties. Moreover, it is clearly unusual among viscoelastic materials in that it has high electrical and thermal conductivity, as well as stability at high temperatures. Third, we were able to show that the material has potential for use as the sensing material in a strain sensor since it presents a linear relationship between resistance and strain. However, the gauge factor value is small (0.43) and efforts to increase it should be considered in future work. These applications are discussed in more detail below.

The material has several advantages for possible use as viscoelastic foam. One drawback to the current generation of polymeric foams is the difficulty of temperature control. For these applications relatively complex composite material foams are now employed to create a pathway for thermal conductivity. Given the far higher electrical and thermal conductivity of CFF relative to polymer-based foams, it should be relatively easy to modify its temperature. For example, the foams can be heated electrically, or temperature maintained simply by contacting the material over only a fraction of the surface area with a heat sink maintained at a constant temperature. Moreover, the range of temperatures at which carbon foams are stable is likely to be far greater than that observed for polymer based viscoelastics, although more study is required to quantify the temperature range at which CFF remain stable while retaining its mechanical properties.

The results of this work indicate that the distinct electrical conductivity of CFF will make it an excellent sensing material in a strain gauge. The key feature of a stain gauge is the availability of a single value electrical signal as a function of strain, a value that reports strain as a “state property”, and is not a function of the history of stress/strain of the material. As shown in Figure 5, the relationship between strain and measured resistance of CFF in simple single axis compression remains linear over many cycles. Resistance is shown to have a single value, within ~2 percent, as a function of strain. Moreover, a linear relationship is observed up to 40% strain, probably because of the large void space fraction (~90%) present in the uncompressed material. That is, as the material is compressed the “void” is squeezed out before the solid fibers are deformed by fiber-to-fiber compression. Thus, resistance can serve as the single value electrical signal required to determine strain over an exceptional range of strain.

Our work shows the CFF performs in a linear fashion at least to a 40% strain. Other carbon-based materials have also been evaluated for use in strain gauges, and these do have relatively high gauge factors, on the order of 20. However, CFF in contrast carbon tubes and fibers need no binders, polymeric matrices or linking additives to form 3D structures of selected shape. Evaluation of the potential use of CFF in multi-axis strain gauges would be appropriate for future studies.

One outcome of this work is the finding that, contrary to an earlier suggestion [49] this material cannot be employed as a pressure gauge. There is a hysteresis clearly observed in the stress-strain curve. This indicates that energy of mechanical deformation observed during the compression leg of a cycle relaxes during the decompression leg and is dissipated as heat.

The hysteresis in stress/strain, but not in stress/resistance provides some insight into the behavior on the microscale. In earlier work it was postulated that resistance changed as a function of strain because the number of contacts between fibers increased with increasing strain, and this would lead to more electrical paths, hence lower resistance. The present work suggests this may not be correct. Indeed, it is generally understood that mechanical relaxation is associated with physical re-arrangement. In the case of fiber foam, that implies that the individual fibers change shape to reduce their mechanical potential energy during relaxation. Certainly this relaxation of many fibers would change the number of fiber-fiber junctions, thus changing resistance. Hence, a change in resistance that matched the magnitude of the change in stress might be expected during transient experiments. This is not observed. Their resistance change, relatively, is much smaller than the stress relaxation. An alternative suggests itself: The conductivity of the individual fibers is changed by strain. That is, there is a relationship between fiber strain and fiber resistance. Specifically, the resistance of each individual fiber decreases as the fiber is shortened. Integrated over a large ensemble of fibers, of many orientations, geometries and sizes, such as that found in a CFF, this leads, on a macroscopic level, to a linear relationship between strain and resistance.

The above suggestion of a relationship between strain and the resistance of individual fibers is in fact consistent with the theory of conductor type strain gauges. Indeed, it is generally understood that single “wires” change resistance because of shape changes, during strain. Broader and shorter wires have lower resistance. In fact, this is the basic physical fact exploited in the design of most strain gauges. Hence, it is a reasonable extension of current understanding of the impact of strain on metal resistance, to apply the same logic to carbon fiber resistance. Moreover, all findings in this work are consistent with this postulate. Finally, there are studies that show the resistances of individual carbon fibers are a function of strain [12]. It is noted this is a reasonable topic for future study.

## 3. Experimental Section

### 3.1. Fiber Growth

**Figure 8.**
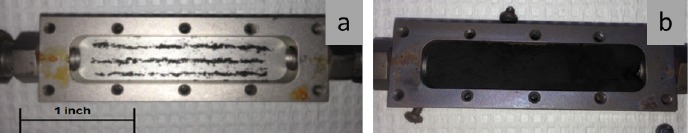
Steel mold and catalyst geometry. Arranging Pd catalyst particles in the mold as shown (**a**), was found to be a necessary part of the protocol required to create, based on visual inspection, homogenous CFF (**b**).

The growth process employed was a variation on the CoFFiN process described elsewhere in which catalyst is arranged in a steel mold and then exposed to a fuel rich mixture of ethylene and oxygen at 550 °C. Previous studies by our team showed that when comparing to other metal catalysts, the most robust fiber structure, grown as a single macroscopic object containing high levels of porosity, are generated from palladium particle catalysts [26]. Thus, in the present work a simple four-step process was followed. First, 20 mg of palladium (Aldrich submicron >99.9%) particles were spread evenly in three equally spaced, 5.72 cm long columns in the bottom of a 304 Stainless Steel mold of dimensions 5.72 cm long × 2.5 cm wide × 0.89 cm height (Figure 8a). This mold chamber was connected to gas flow system (0.64 cm OD stainless steel tubing) capable of simultaneous control of the four gases using an MKS 647a flow controller. The mold chamber itself was positioned at the center of a 45.72 cm long × 5.08 cm diameter single zone Lindberg/Blue Mini-Mite furnace tube furnace with a maximum temperature capability of 1200 °C. Second, the mold/catalyst was thoroughly flushed with UHP Nitrogen. Third, the temperature was raised (25 °C/min) to 550°C, and the reactive gas mixture, composed of ethylene, oxygen and nitrogen diluent, introduced. As earlier work showed that gas residence time and composition strongly impacted the growth rate, the flow rates were carefully adjusted to be ethylene 15 SCCM/oxygen 15 SCCM and nitrogen 100 SCCM. Finally, after two hours and forty five minutes ethylene, oxygen and the furnace were turned off, and the system allowed to cool. The single block of CFF (Figure 8b) was then removed from the mold, and cut into smaller pieces for mechanical/electrical testing, observation using SEM or thermal testing in a TGA/DSC.

### 3.2. Samples Characterization

In order to examine the microstructure of the carbonaceous specimens the samples were attached to a standard aluminum holder using carbon tape, placed in vacuum overnight and introduced in the chamber of a Zeiss Neon 40 High Resolution Scanning Electron Microscope (SEM) (Zeiss, Oberkochen, Germany). Images were acquired at diverse magnifications while microscope was operated at 10 or 20 kV. Energy Dispersive Spectroscopy (EDS) experiments were conducted in conjunction with the SEM using the EDAX equipment with an Apollo 10 silicon drift detector (SDD). Data was collected and analyzed using Genesis Spectrum software.

A MTI Instruments MTII/Fullam SEM tester (MTI Instruments, Albany, New York, NY, USA), with a maximum load capability of 4500 N was used to apply diverse compressive loads to the samples inside the SEM chamber and observe microstructural changes *in situ*.

A Netzsch STA 449 FE Jupiter (Netzsch, Gerätebau, Germany), operated in a TPO mode, was used to study the thermal stability of the samples. The samples were exposed to an Ar/O_2_, 80%/20% atmosphere, total flow of 120 mL/min, from RT to 1000 °C at a heating rate of 10 °C/min.

Brunauer Emmet Teller (BET) surface area analysis was performed employing a Quantachrome Nova 4200 (Quantachrome, Boynton Beach, FL, USA). A 300 °C degas step was conducted prior to the analysis; samples were then allowed to cool down to room temperature and then transferred to the analysis station. The measurements were done using nitrogen atmosphere.

The relative density of the product was calculated as the ratio of the mass of a portion of the sample with respect of its volume. The volume of the material was measured using a graduated cylinder filled with DI water, immersing the sample on it and determining the volume of water displaced.

The contact angle of a water droplet in the surface of the as prepared solid was measured from images taken with the CCD camera of a digital microscope. The water was dispensed using a micro-syringe and the contact angle evaluated by fitting the drop profile according to the Young-Laplace method using the software Image J for image processing [50]. Mineral oil, Pfeiffer D-35614 Asslar Oil P3 (Pfeiffer, Asslar, Germany), was used to observe the fiber structure interaction with a non-polar substance.

### 3.3. Mechanical and Electrical Testing

**Figure 9.**
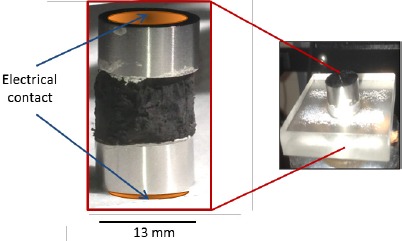
Anvils and sample placement for simultaneous mechanical and electrical tests. Sections of the carbon fiber based material were cut to the same diameter than the anvils, placed inside a Plexiglas cavity to avoid changes in cross sectional area during measurements.

In order to mechanically characterize the carbon fiber samples, several different test conditions were employed: dynamic; with variable loads changing at different rates over time, and transient; with static loads maintained as a function of time. To ensure that the samples only changed dimensions in the direction of applied force and that their top and bottom maintained a constant area during resistivity measurements, the highly porous samples were cut to cylindrical shapes. Those cylindrical shapes were made to match the size and shape of two 13 mm stainless steel anvils that served as platens to compress the sample and as electrical contacts. A Plexiglas mold with a cavity of 1.3 cm diameter was used to maintain the sample cylinder and anvils aligned during tests (Figure 9). The anvils closely fit the mold gap without touching the Plexiglas, therefore creating no friction against it. There was no space for the carbon sample to expand in the lateral direction during compression; hence, tests in this configuration were denominated as constrained.

An Instron 5942 with a 100 N load cell (Instron, Norwood, MA, USA), configured to study the material in compression, was employed for all macroscopic tests. The compressive force was selected to remain between 10 and 90 N. The lower limit was selected in order to prevent the carbon nano-fiber from losing contact with the anvil while the upper limit was chosen to prevent reaching the 100 N automatic cutout limit on the load cell used in the test.

The system was organized to allow simultaneous measure of the electrical, specifically resistance, and mechanical properties. This required creating an electrical connection, using copper tape, to each anvil. As control studies showed zero resistance when the anvils touched, the resistance measured was simply that of the CFF between them. The resistance was measured using an Agilent 34410A 6 ½ Digital Multimeter (Agilent, Santa Clara, CA, USA) with software that permitted direct connected to a PC computer and data download directly to a spreadsheet.

Transient tests were performed at diverse levels of constant stress and the change in strain as a function of time recorded. For simplicity only the results for samples held at 90 Newton over 72 h were included in this manuscript. Cyclic tests, generally known as dynamic tests, were performed by linear change in strain as a function of time between two stress limits. Two strain rates were employed: 0.01 and 0.05 mm/s. Load limits for the cycles were within one of the following values: 10 to 40, 40 to 90, or 10 to 90 N.

## 4. Conclusions

Carbon fiber foams (CFF) were grown using the Constrained Formation of Fibrous Nanostructures process (CoFFin). The outcome of the growth process suggests these foams can be grown to any desired shape in a mold. For the first time the viscoelastic properties of the foam were demonstrated. Moreover, microscopic evaluation showed the foams to have a distinct microstructure, intertwined nano-fibers, for a viscoelastic material. As with many other recently discovered carbon structures, this foam has distinctive properties that imply improved performance for various uses. The high conductivity, low specific gravity, high temperature stability and hydrophobicity also suggest this material would be superior to existing closed cell viscoelastics for many applications: shock absorber foam, sensing element, electrode material, filter or absorbent membrane, and low drag surface, among others.
